# Structural Evolution of Ultrathin SrFeO_3−δ_ Films during Oxygen Evolution Reaction Revealed by *In Situ* Electrochemical Stress Measurements

**DOI:** 10.1021/acsaem.3c01805

**Published:** 2023-11-16

**Authors:** Emily Marquez, Kim Hong Keu, Andrea Nelson, Benjamin M. Lefler, Steven J. May, Hadi Tavassol

**Affiliations:** † Department of Chemistry and Biochemistry, California State University, Long Beach, California 90840, United States; ‡ Department of Physics and Astronomy, California State University, Long Beach, California 90840, United States; § Department of Materials Science and Engineering, Drexel University, Pennsylvania 19104, United States

**Keywords:** iron perovskites, OER, iron oxides, surface stress analysis, in situ structural evolutions

## Abstract

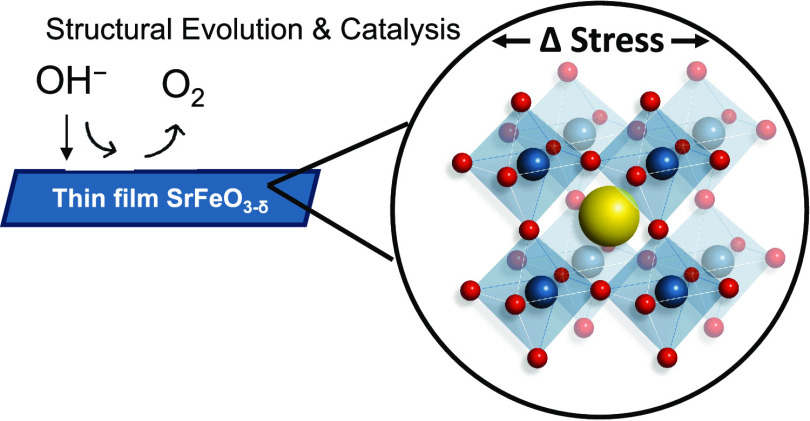

We report the electrochemical stress analysis of SrFeO_3−δ_ (SFO) films deposited on Au substrates during oxygen evolution reactions (OERs). Our *in situ* analysis of Au reveals conversion reactions from Au to Au(OH)_3_, AuOOH, and AuO_*x*_ during the OER. Au reactions cause a monotonic compressive stress on surfaces assigned to the formation of Au hydroxides and oxides. Electrochemical stress analysis of SrFeO_3−δ_/Au shows a dramatically different behavior during the OER, which we attribute to structural evolutions and conversion reactions, such as the conversion of SFO to iron (oxy)hydroxides. Interestingly, electrochemical stress analysis of SrFeO_3−δ_/Au shows a tensile trend, which evolves with cycling history. Electrochemical stress analysis of SFO films before the onset of the OER shows *in situ* changes, which cause tensile stresses when cycling to 1.2 V. We attribute these stresses to the formation of Fe^2+δ^O_δ_(OH)_2−δ_ (0 ≤ δ ≤ 1.5)-type materials where δ approaches 1.5 at higher potentials. At potentials higher than 1.2 V and during OER, surface stress response is rather stable, which we assign to the full conversion of SFO to iron (oxy)hydroxides. This analysis provides insight into the reaction mechanism and details of *in situ* structural changes of iron perovskites during the OER in alkaline environments.

## Introduction

Oxygen evolution reaction (OER) is the catalytically challenging half-reaction of several processes important in the sustainable energy conversion and storage cycles, such as water splitting and CO_2_ reduction.^1,2^ OER in alkaline environments, 4OH^–^ → O_2_ + 4e^–^ + 2 H_2_O, shows high overpotentials even on most active catalysts.^3−6^ Nature uses photosystem II, which contains earth-abundant transition-metal (*i.e*., Mn) active sites for oxygen evolution reaction in plants.^7,8^ Iron–sulfur (Fe–S) clusters are also ubiquitous in natural systems.^9^ 3d transition-metal oxides of Co, Fe, Ni, and Mn have been shown to be active for OER electrocatalysis.^10−14^ These materials are believed to undergo *in situ* transformations during OER. *In situ* formation of oxyhydroxides and double hydroxides has been linked to higher activities of several transition-metal oxides.^1,11,15−17^ NiFe (oxy)hydroxides are the most active electrocatalyst in alkaline media.^18^ However, details of *in situ* transformation of active sites of transition-metal (oxy)hydroxides remain unknown.^12,16,18^

Fe doping and/or contamination in Ni(oxy)hydroxides has also been shown to improve OER activity.^3,15,16,19,20^ Explaining the differences in the activity of Fe-only and Fe–M (M=Ni, Co)-based catalysts remains challenging.^2,21−24^ Activity of iron perovskites toward OER electrocatalysis also shows large variation.^23,24^ Particularly, SrFeO_3−δ_-type perovskite and double perovskites have shown a wide range of activity in alkaline solutions.^13,25,26^ Here, we use thin films of SFO deposited on Au as a model system to study surface interactions and structural changes during the OER using *in situ* electrochemical stress measurements. Our analysis shows that ultrathin SFO films undergo structural evolutions during OER in alkaline conditions. The *in situ* stress analysis of thin films enables us to monitor surface structural evolutions, which are often not visible when bulk oxide samples are used or oxide materials are probed by bulk characterization methods.

## Results and Discussion

### Electrochemical Stress of Au during OER in Alkaline Solutions

**Figure 1:**
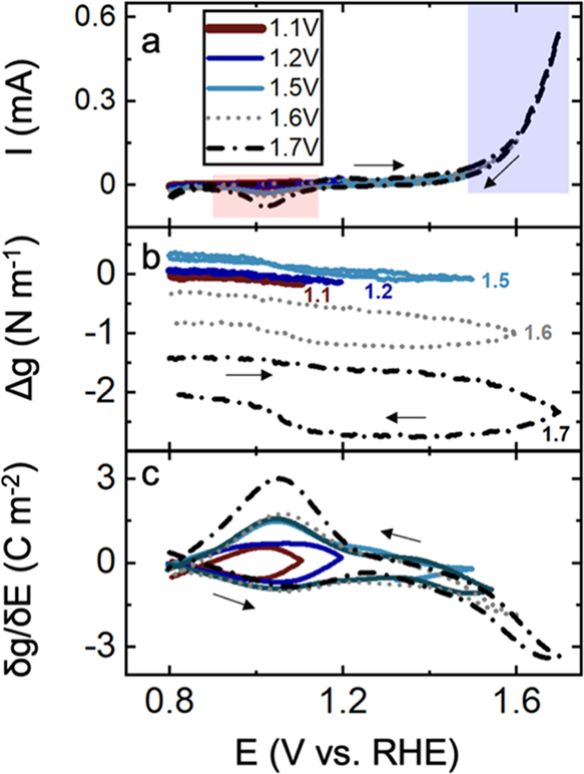
(a) Cyclic voltammetry of a Au film in 0.1 M KOH in the 0.8–1.7 V potential range at a scan rate of 20 mV s^–1^. The voltage range was increased by 50 mV increments after each electrochemical stress measurement starting from 1.05 to 1.7 V where the results from continuous cycling are shown. The inset and legends show the high potential limit for each cycle. (b) Corresponding *in situ* stress response and (c) first derivative of stress with respect to the potential. Oxidative (blue) and reductive (red) features are highlighted.

Figure 1 shows the electrochemical stress analysis of a textured Au film deposited on glass with a Cr adhesion layer (as further explained in the Experimental Section) in an Ar-saturated 0.1 M KOH solution. Figure 1a shows the cyclic voltammetry of the Au in 0.1 M KOH solution, which agrees with previous reports of electrochemical analysis of Au surfaces in alkaline environments.^27−30^ The onset of OER is visible at 1.4 V on Au surfaces, which is consistent with previous reports.^27,31^ The small oxidative feature at 1.2 V is associated with the surface adsorption of OH^–^ and the formation of Au(OH)_*x*_.^27,31,32^ At more positive potentials, AuO_*x*_ features are visible, which are followed by the anodic current associated with OER. The following occurs during oxidation^30,31,33^
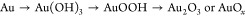
During the reductive sweep, the reductive feature at 1.05 V is assigned to the reduction of Au(III) to Au(I).^7,9^ Hydroxide desorption also occurs during this potential region.^27,30,31^

Figure 1b shows the corresponding stress measurement. Stress measurements using the setup explained in the Experimental Section report the ensemble stress difference between the surface of interest and an inert surface, here between Au and the backside glass.^34−37^ If the active thickness of the interface is known, then stress in units of pascal (Pa) can be calculated by dividing stress-thickness by the active thickness of the film. The stress response of Au in 0.1 M KOH solutions (Figure 1b) shows that as potential is scanned to more positive values, *i.e*., the anodic sweep, the stress response shows an overall compressive trend indicated by a negative Δ*g* value. A compressive stress refers to cases where the surface experiences compression, such as during insertion of a metal cation in a host, for example, electrodeposition of Cu/Au and Li/Au. Tensile response refers to the tension generated on a surface, for example, during Cu and Li stripping from the Au surface.^36−38^ The stress response shows a less pronounced change when the high potential limits are below 1.2 V, which corresponds to the thermodynamic water oxidation potential. However, as the positive potential limit increases, the magnitude of the compressive stress also shows an increasing trend. During the cathodic sweep, the stress response shows an overall tensile trend as revealed by a decreasing magnitude of the negative Δ*g*. For cyclic voltammetry analyses with less positive high potential limits, *i.e*., less than 1.2 V, the tensile stress removes the compressive stress observed during oxidation, and hence, no residual stress is observed. As the potential is swept to higher values with increasing current densities, an increasing residual compressive stress is observed. The residual compressive stress points to a change in the composition of Au surfaces during OER.^27^ It should be noted that the compressive stress observed during the anodic sweep at potentials higher than 1.5 V is due to the OER. However, due to the slow kinetics on surfaces studied here, the formation of an O_2_ bubble is not expected. The formation of bubbles will cause disruptions in the laser reflection, which are evident in the stress response. In the stress response of Au and SrFeO_3−δ_/Au presented here, such effects are not present.

Figure 1c shows the derivative of stress with respect to potential (δ*g*/δ*E*, C m^–2^), which has the units of surface charge and correlates with charged species at the surface.^39−41^ Interestingly, δ*g*/δ*E* is dominated by the features present at 1 V, which are likely due to OH^–^ adsorption on Au surfaces, and Au(OH)_*x*_ formation evident by the small feature at 1.2 V in the cyclic voltammetry (Figures 1a and S1). At more positive potential at 1.4 V, the δ*g*/δ*E* shows a dominant feature corresponding to AuO_*x*_ formation and OER.

**Figure 2:**
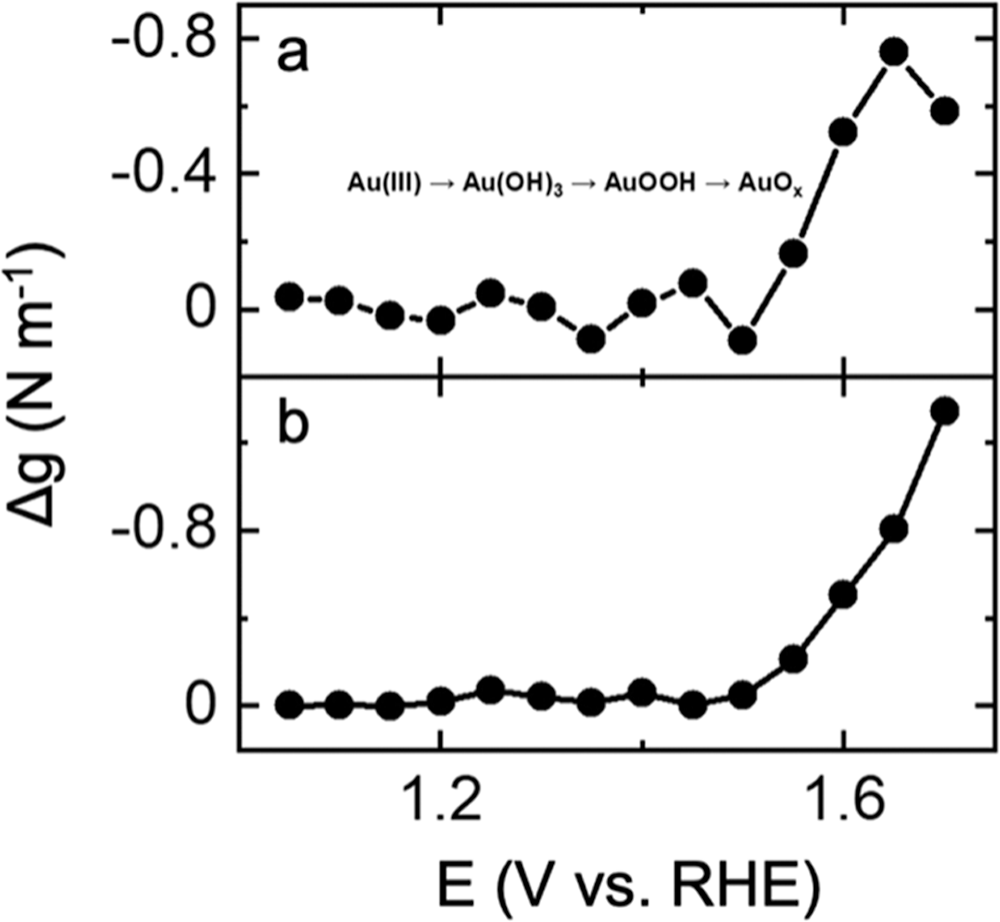
(a) End-of-the-cycle residual stress and (b) maximum hysteresis observed during the electrochemical cycling of Au in alkaline solutions as a function of the high potential limit.

Figure 2a shows the end-of-the-cycle residual stress as a function of the positive potential range, which mostly exhibits an overall compressive trend (increasing negative magnitude of Δ*g*) assigned to irreversible Au oxidation. In fact, the maximum hysteresis observed during the electrochemical oxidation of Au is compressive for all voltage ranges (Figure 2b). The maximum hysteresis is observed at 1.7 V, with a stress-thickness of *ca*. 1.3 N m^–1^, which corresponds to 5 GPa stress, assuming that the OER is limited to the topmost layer of the Au surface.

Interestingly, when the higher potential limit is in the range of 1.1–1.5 V, variations in the trend of the residual stress are evident. The variations may be due to the stepwise formation of Au(OH)_*x*_ and AuOOH and eventually AuO_*x*_, which shows a clear residual compressive stress response. Such residual stresses and hysteresis are likely related to the structural and volume changes associated with Au oxidation in different potential regions.

**Figure 3:**
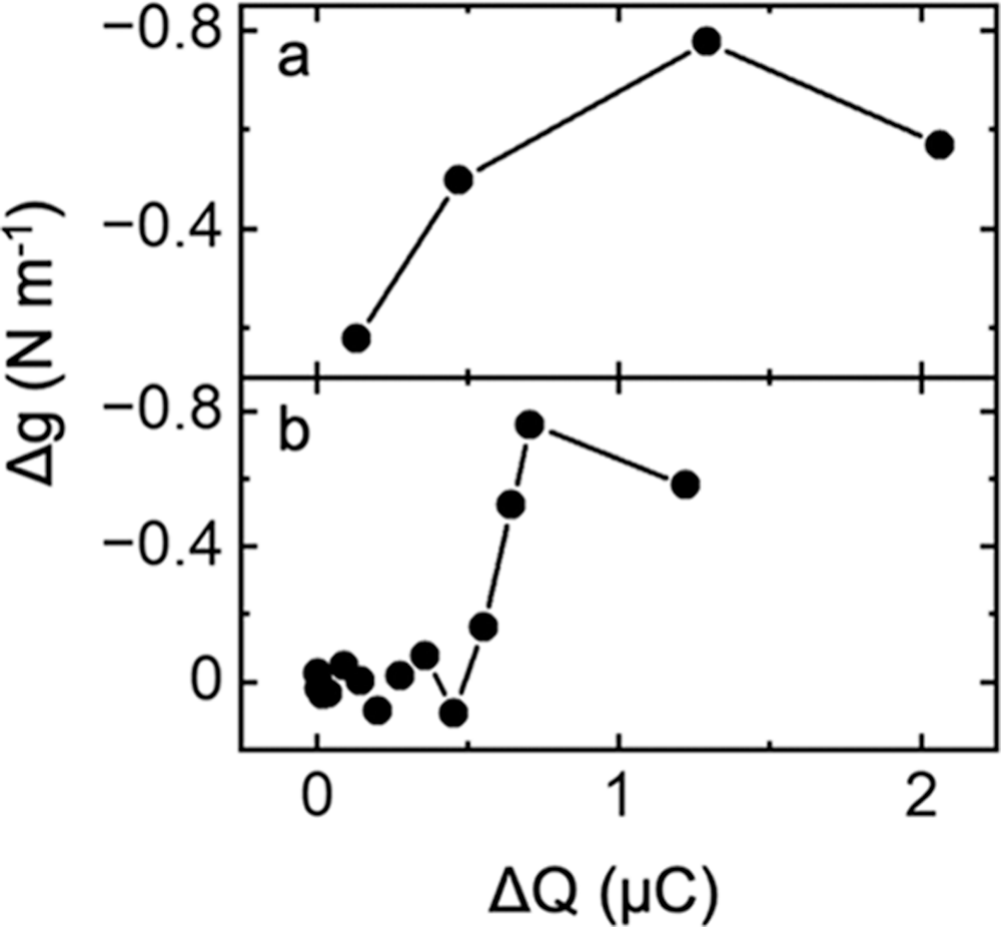
End-of-cycle residual Δ*g* (end-of-cycle residual stress at 0.8 V vs RHE) vs. Δ*Q* for Au in 0.1 M KOH. (a) Δ*Q* acquired by integrating the oxidative features assigned to the OER starting at 1.5 V, while (b) Δ*Q* acquired by integrating the reductive feature at *ca*. 1 V.

Figure 3 shows the end-of-the-cycle residual stress (at the starting potential of 0.8 V) as a function of the electrochemical charge associated with two main oxidative and reductive features observed in the voltammetry (Figure 1, highlighted red/blue). Figure 3a shows the stress response Δg vs integrated charge (Δ*Q*) of the oxidative feature observed during the OER as the electrode potential is cycled to potentials more positive than 1.5 V (*ca*. 1.5–1.7 V). Figure 3b shows the integrated charge from the reductive feature at *ca*. ∼0.95 V to 1. Interestingly, the associate charge (Δ*Q*) from the oxidative feature correlates with the residual stress observed on Au surfaces, which confirms that the residual stress is due to the irreversible oxidation of Au products at higher potentials.

Our analysis indicates that when Au oxidation is terminated in any of Au(OH)_*x*_ or AuOOH, then the residual stress associated with this process upon reduction shows tensile features. However, when Au is fully oxidized and is in the OER process, the reduction of the surface is not fully reversible; hence, the residual stresses associated with these potential regions are compressive.

### Electrochemical Stress of SFO/Au Films during the Pre-OER Region

Here, we use thin films of SrFeO_3−δ_ on Au substrates to study the oxide reactions during OER. SrFeO_3−δ_ and Fe oxide-based catalysts are attractive for different reactions, *e.g*., OER and methane oxidation.^42^ SFO-type catalysts for oxygen evolution reactions have shown a range of activity and stability in alkaline environments. There seem to be significant differences in the observed activity of similar SFO-type catalysts.^10,43^ However, the detailed *in situ* evolution of these structures during the OER remains unknown. We use electrochemical stress measurement of SFO/Au films to probe the *in situ* evolution of these structures. The stress analysis of Au in alkaline media presented in Figure 1 serves as a baseline for this analysis.

**Figure 4:**
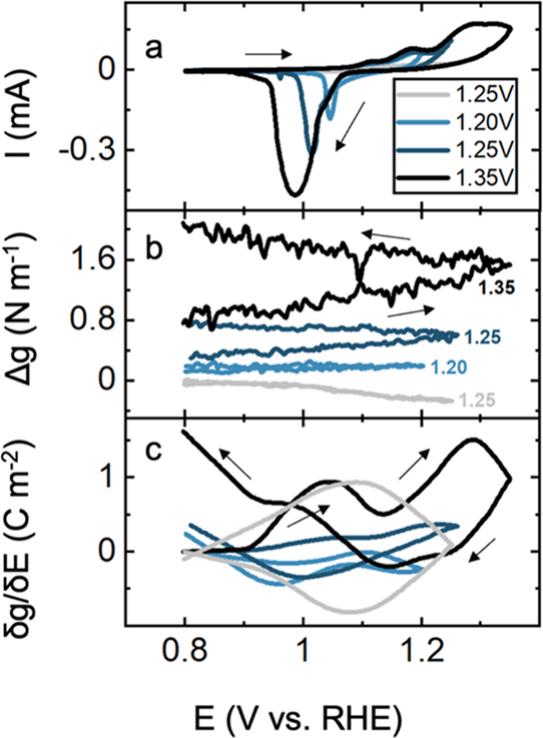
(a) Cyclic voltammetry of SrFeO_3−δ_/Au in 0.1 M KOH in the 0.8–1.35 V potential range, *i.e*., *pre*-OER region at a scan rate of 20 mV s^–1^ where different continuous electrochemical cycles are shown. The inset and legends show the high potential limit of each cycle (b) corresponding to *in situ* stress response and (c) first derivative of stress with respect to the potential. The data shown in light gray (b,c) was obtained from Au.

Figure 4 presents the electrochemical stress analysis of SFO/Au thin films. Figure 4a shows cyclic voltammetry analysis of SFO/Au films at different potential ranges from 0.8 to 1.4 V, which are dominated by features assigned to surface oxidation and pre-OER (more potential ranges are included in the SI). The cyclic voltammetry of SFO/Au is consistent with thin films of SFO-type materials and is different from the voltammetry of Au substrate presented above.^10,44,45^ Early oxidative features on SFO/Au are evident at *ca*. 1.1 V, and as the potential moves to more positive values, multiple oxidative features are present. As potential is decreased, a dominant reductive feature in the potential range of 0.9–1.1 V is present, which moves to lower potentials upon cycling to higher potentials during the positive-going scan. Such changes in the voltammetry of SFO/Au is likely due to the structural evolutions and/or oxide conversion reactions. This structural evolution is further displayed in Figure S3 in which the high potential limit is changed to more positive potentials starting from 1.2 to 1.65 V, showing constant change in voltammetry, indicating an evolving oxide material. The reductive feature at 0.9–1.1 V, which shows an increasing current with cycling to a more positive potential, also exhibits a constant shift to more negative potentials. Such changes in oxide electrode materials have been previously observed, *e.g*., with SnO_*x*_ oxide conversion reactions.^38,46−48^ This suggests that Fe may also exist outside the perovskite structure, particularly at the surface. Considering iron’s Pourbaix diagram,^49^ at low potential relevant to HER, iron is in Fe(OH)_2_, and at more positive potentials, Fe(OH)_2_, FeOOH, Fe(OH)_3_, and FeO_*x*_ are likely to form. Assuming the iron is released from the perovskite framework, we use the general formula of Fe^2+δ^O_δ_(OH)_2−δ_ (0 ≤ δ ≤ 1.5),^13^ where δ increases with increasing potential toward OER.^50^

Figure 4b shows the corresponding stress responses of SFO/Au films. Initially, stress response seems to be flat for high potential limits of <1.2 V. Stress response shows a consistent tensile pattern with cycling to more positive potentials (>1.2 V). For these potentials, residual tensile stresses are also present at the end of each electrochemical cycle as high potential limits move from 1.2 to 1.35 V. This tensile stress is notably different from the compressive stress observed on Au substrates (light gray), which indicates that the SFO top layer is controlling the stress response.

Tensile stress during the OER has been observed on other surfaces, *e.g*., Ni and Co in alkaline environments.^27^ The tensile stress on Ni and Co was assigned to the formation of MOOH (M=Ni and Co) with smaller M–M and M–O bond lengths.^11,14,19,27,51^ Prior to electrochemical cycling, on the surface of SFO/Au electrodes, iron is in the SrFeO_3−δ_ perovskite structure where δ is the oxygen deficiency, a nonzero value at room temperature.^11,14,19,27,51^ After electrochemical cycling and because of surface interaction with a pH 13 solution, we assign the stress behavior observed here to the formation of Fe^2+δ^O_δ_(OH)_2−δ_ (0 ≤ δ ≤ 1.5).

During OER on oxide surfaces, M–M and M–O bond lengths particularly during oxide conversion reactions^11,14,19,27,48,51^ most likely determine the nature of stress response (*e.g*., compressive or tensile). Previous studies have shown that different transition metals have varying stress responses during OER; for example, Au and Ir^27,52−54^ have shown compressive response, while Co and Ni^11,14,19,51^ have shown tensile stress during oxygen reactions in alkaline environments.

Previous literature has reported that the Fe–O bond length for Fe(OH)_2_ is ∼2.11 Å,^17,55^ and Fe–Fe bond length ranges from 2.99 to 3.07 Å.^56^ The Fe–O bond length in FeOOH is in the range of 1.95–2.07 Å during the OER region.^13^ Fe_2_O_3_ has Fe–O bond lengths of 1.98 and 2.13 Å in a lattice structure. However, these bond lengths vary with oxygen content. In any case, possible changes in surface bond lengths are due to conversion reactions primarily at the surface, and the continuous tensile stress response in the SFO/Au system agrees with a constant contraction of the structure due to the formation of iron oxyhydroxides according to the general formula of Fe^2+δ^O_δ_(OH)_2−δ_ (0 ≤ δ ≤ 1.5)^13^ with higher δ as potential moves to more positive potentials.^16,57^ This observation is consistent with the tensile stress observed in several surfaces forming MOOH.^27,57^ The stress responses in this region are likely also controlled by hydroxide intercalation within the perovskite structure.^58−60^ It should be noted that tensile stress observed here does not show any evidence of a sudden phase change, occurring at a narrow potential range, but rather a continuous potential-dependent conversion of the material.

**Figure 5:**
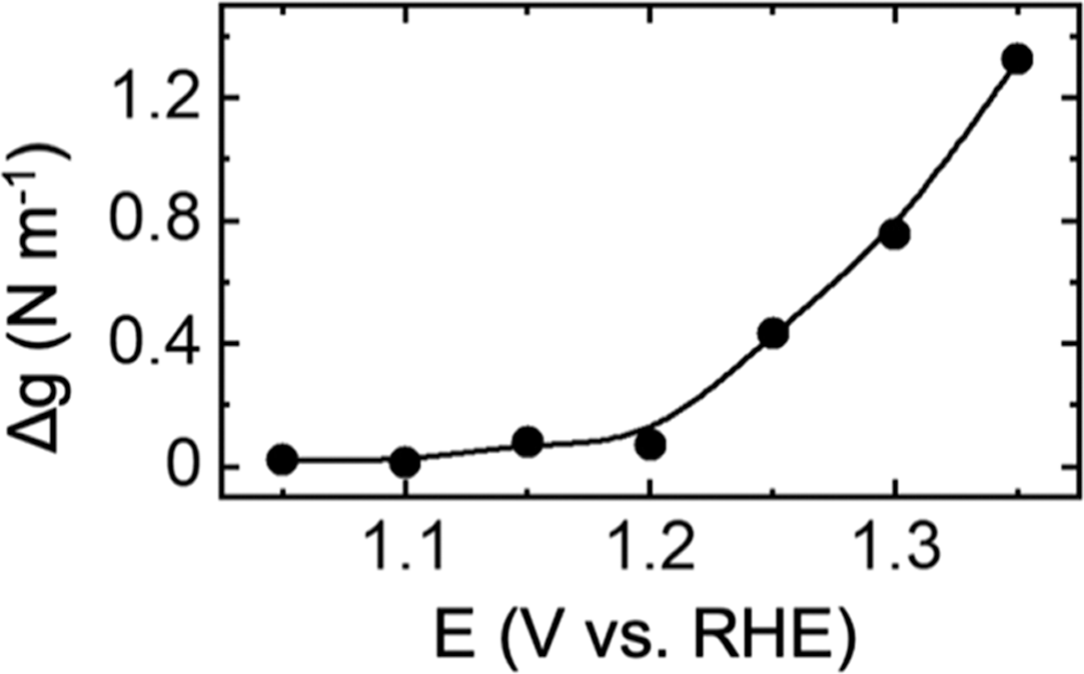
End-of-cycle residual Δ*g* (tensile stress as indicated by the positive sign) as a function of the high potential limit of voltammetry is shown in Figure 4a.

As mentioned, cycling to more positive potentials results in residual tensile stresses at the end of each electrochemical cycle. Figure 5 shows the end-of-the-cycle stress as a function of the high potential limit of voltammetry. The residual stress remains negligible for potentials of ≤1.2 V; however, at higher potentials, residual tensile stress correlates with potential as further oxidation occurs on the surface.

As shown in the voltammetry of Figure 4a (and Figure S3), the current and charge associated with the reductive feature at *ca*. 0.9–1.1 V also increase when potential is cycled to more positive values (0.9–1.35 V).

**Figure 6:**
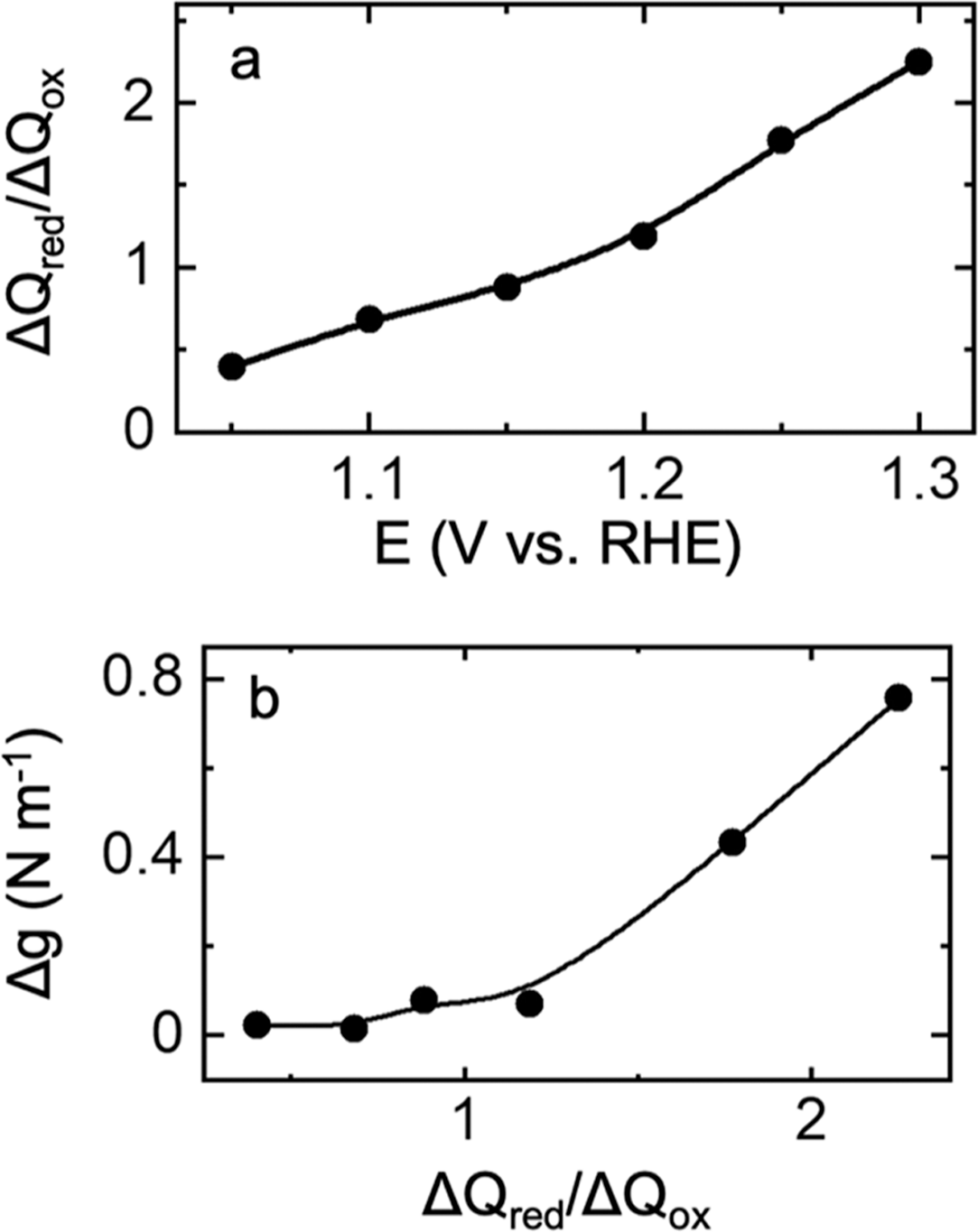
(a) Integrated Δ*Q* reductive peak versus oxidative peak vs. high potential limit. (b) Residual stress with respect to charge ratio, Δ*Q*_red_/Δ*Q*_ox_, for SrFeO_3−δ_*/*Au in 0.1 M KOH.

Figures 5 and S5 reveal two distinct regions of structural or compositional changes. In the first region of ≤1.2 V, upon scanning to higher potentials, more charge in the reductive feature at 0.9–1.1 V does not result in higher residual stress, pointing to primarily reversible conversion reactions during oxidation. In the second region, >1.2 V, the residual stress shows a positive correlation with the high potential limit and the charge of the 0.9–1.1 V reductive feature, pointing to oxidative processes that cause irreversible tensile changes. Figure 6a shows Δ*Q*_red_/Δ*Q*_ox_ associated with the oxidative features at potential >1.1 V and the single reductive feature at 0.9–1.1 V as a function of the high potential limit, which shows that at higher potentials irreversible oxidations occur where Δ*Q*_red_/Δ*Q*_ox_ > 1. Figure 6b shows Δ*Q*_red_/Δ*Q*_ox_ as a function of residual stress, which correlates with the irreversibility of oxide reactions. When Δ*Q*_red_/Δ*Q*_ox_ is close to unity, residual stress approaches zero, and as more irreversible oxide reactions occur, *i.e*., increasing the Δ*Q*_red_/Δ*Q*_ox_ ratio, residual tensile stress increases. These irreversible oxidative features evident from residual stress and charges associated with voltammetric features further support conversion reactions and structural changes of the SFO surface to Fe^2+δ^O_δ_(OH)_2−δ_ (0 ≤ δ ≤ 1.5)-type materials, where more positive potentials result in an increased residual tensile stress.

**Figure 7:**
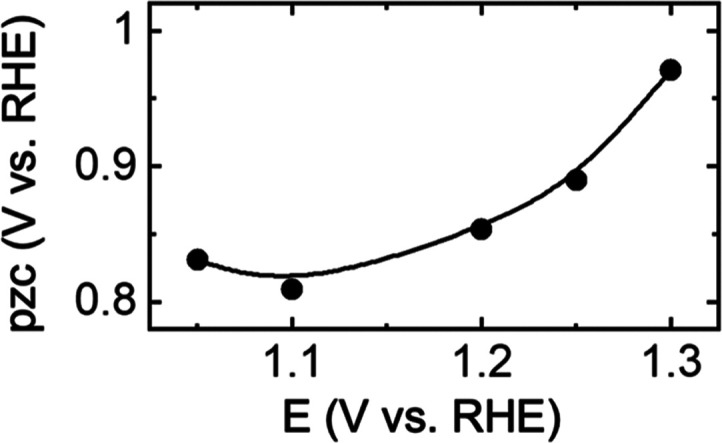
Estimated potential of zero charge (pzc) from derivative of stress with respect to potential as a function of the high potential limit.

Figure 4c shows the derivative of stress with respect to potential (δ*g*/δ*E*) as a function of potential. Interestingly, unlike the behavior observed for Au, where δ*g*/δ*E* mimicked the voltammetry, here both the high potential limit and cycling history change the δ*g*/δ*E* response. This observation further points to structural and compositional changes in SrFeO_3−δ_ during the pre-OER region. Since δ*g*/δ*E* has the units of surface charge, it may reflect charged species on the surface, we use the crossing points of positive- and negative-going scans, which are generally located at δ*g*/δ*E* = 0 to estimate the potential of zero charge (pzc). Figure 7 shows the pzc estimated from the derivative of stress with respect to potential as a function of the high potential limit. Point of zero charge, the pH with total zero surface charge for an unpolarized surface, has been reported in the range of 5–9 for iron oxides.^61,62^ This implies that in alkaline conditions used here, iron oxide-type materials have a net negative charge under unpolarized conditions.

Here, using δ*g*/δ*E*, we estimate a pzc, the potential at which the total charge of the surface is zero, of 0.84 when the potential is cycled to 1.05 V. Estimated pzc shows an increasing trend with increasing potential limit. These increasing pzc values are consistent with the absorption of OH^–^ from solution and more oxidation on the surface resulting in the formation of iron (oxy)hydroxides, Fe^2+δ^O_δ_(OH)_2−δ_ (0 ≤ δ ≤ 1.5) at higher potentials.

**Figure 8:**
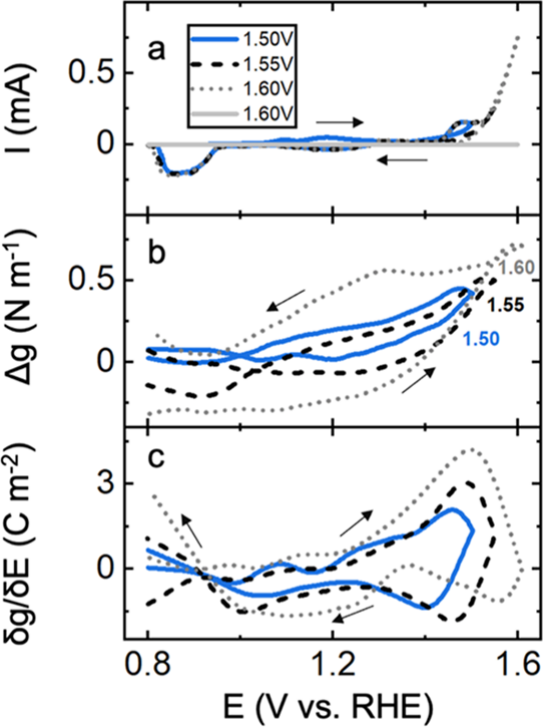
(a) CV of SrFeO_3−δ_/Au in 0.1 M KOH in the voltage range from 0.8 to 1.6 V where cycles were measured consecutively (Au cyclic voltammetry is added as a reference in light gray). (b) Corresponding stress measurements and (c) the corresponding first derivative of stress with respect to the applied potential.

Figure 8 shows electrochemical stress analysis of SrFeO_3−δ_/Au at a more positive potential region (*ca*. 1.4 V) during oxygen evolution reaction. Figure 8a shows cyclic voltammetry analysis of the SFO/Au film, which is consistent with previous reports of SFO during OER.^10,23,24^ Cycling to more positive potentials results in higher oxidative current associated with the OER. However, the cathodic feature at *ca*. 1 V remains constant. This implies that the more oxidative current observed at higher potentials is due to OER. This is contrary to what we observed during the pre-OER region where the reductive feature at 0.9–1.1 V evolved with cycling to more positive potentials. Figure 8b shows the corresponding electrochemical stress response with a tensile stress evident during the oxidative scan and a compressive stress shown during the reductive scan. Interestingly, electrochemical stress in this potential range does not show significant residual stresses and the overall behavior remains relatively stable, where tensile stresses developed during oxidation are removed upon reduction, even with cycling to more positive potentials. We can consider the active thickness of the material to compare the stresses experienced at the SFO surface at different potential regions. In the pre-OER region (cycling to 1.2 V), assuming that only one layer of SFO is involved in the reactions, the observed structural changes described above cause a 3 GPa stress on the surface, which we attributed to the formation of Fe^2+δ^O_δ_(OH)_2−δ_ (0 ≤ δ ≤ 1.5). During OER (cycling to 1.6 V), the stress analysis shows that SFO is likely fully converted to iron (oxy)hydroxides, where a stable stress response is evident. Assuming that the active thickness of the film is the entire thickness of the SFO (∼7 nm), the structural change during this potential region causes a stress of 3 MPa. These values represent the range of stresses induced on the surface depending on the active thickness undergoing structural evolution.

Figure 8c shows the derivative of stress with respect to the potential (δ*g*/δ*E*) as a function of potential. The derivative response also shows major features during the oxygen evolution reaction and remains stable with more cycling to higher potentials. Interestingly, the crossing points of positive- and negative-going scans at δ*g*/δ*E* = 0 remain constant with cycling to more positive potentials. The overall stable behavior observed in the electrochemical stress analysis during the OER is assigned to the formation of iron (oxy)hydroxides Fe^2+δ^O_δ_(OH)_2−δ_ (0 ≤ δ ≤ 1.5) following the pre-OER region. Our stress analysis provides evidence for surface structural transformations of SFO during the OER; however, further analyses of *in situ* material transformation are needed to provide definitive proof for surface transformations and formation of iron oxyhydroxide phases and to resolve the fate of surface and subsurface Sr.

## Conclusions

Our electrochemical stress analysis of Au and SrFeO_3−δ_/Au samples shows characteristic features corresponding to oxide formation and phase changes during the OER. On Au surfaces, our analysis shows end-of-cycle irreversible compressive stresses during the OER, which corresponds to the formation of hydroxide, (oxy)hydroxide, and oxides of Au. Au reactions show similar stress behaviors over long-term cycling and when cycling to higher potentials. Electrochemical stress analysis of thin film SrFeO_3−δ_/Au shows evolving tensile stresses initially in the potential range of 0.8–1.3 V, which are attributed to the formation of Fe^2+δ^O_δ_(OH)_2−δ_ (0 ≤ δ ≤ 1.5) where δ increases at higher potentials. Upon cycling to higher potentials of 1.6 V, and completion of conversion reactions, electrochemical stress in the SFO shows a more reversible behavior. Our analysis provides insight into the SFO-type oxide conversion reactions occurring during the OER in alkaline media, pointing to a stepwise structural evolution of these materials into iron (oxy)hydroxide-type materials.

## Experimental Section

### Deposition of Au

Au cantilevers were made using an Angstrom Engineering Covap II. The glass substrates (Fisherbrand, 1.0 mm thick) were first cleaned with a sequence of acetone, isopropyl alcohol, and methanol. The thermal evaporator was set to deposit a 10 nm Cr adhesion layer, followed by a 50 nm Au layer.

SrFeO_3–*x*_ (*x* ∼ 0.5) films were deposited onto the Au/glass substrates using oxide molecular beam epitaxy, using deposition conditions reported in ref (63). Sr and Fe were codeposited from effusion cells at a growth rate of approximately 0.4 nm/min.^63^ The substrate growth temperature was held at ∼600 °C, while O_2_ was delivered through a capillary tube aimed at the substrate, resulting in a chamber pressure of 3 × 10^–6^ Torr during deposition. The thickness of the SFO film is approximately 7 nm. Following growth, the film was subjected to an oxidizing anneal in a 5:95 mixture of O_3_/O_2_ for 1 h at approximately 150 °C to decrease *x*.^63^

The electrochemical experiment was performed in a 0.1 M KOH buffer solution. 0.1 M H_2_SO_4_ was prepared from a stock solution (93–98% purity) and deionized water (Milli-Q).

### Electrochemistry

Electrochemical stress cantilevers (working electrodes) were rinsed with Milli-Q water. A three-electrode cell setup was used with Pt wire (counter electrode) and Ag/AgCl (reference electrode). Hydrogen calibration was conducted before the cyclic voltammetry to convert measurements to potential versus reversible hydrogen electrode (V vs RHE). Electrolyte solution (0.1 M KOH) was purged under Ar for 30 min. All solutions were made using high-purity materials and Milli-Q water (with a conductivity of <18 μS). We also use “leakless” Ag/AgCl. All solutions were purged with Ar gas prior to experiments. All glassware and cells were cleaned using concentrated sulfuric and nitric baths and rinsed with boiling water to minimize trace metal impurities. All measurements were conducted in ambient conditions. Data was recorded using CH Instruments potentiostat.

### Stress Measurements

Stress calculations measure the change in the curvature of thin glass cantilevers using an optical laser setup, which has been previously described.^35,38,64,65^ The He–Ne laser scanning line is modulated onto a position-sensitive detector (PSD) with an oscillating mirror. The cell was sealed with a quartz optical window, and the cantilever was mounted using a 3D-printed cap with a Au contact. The electrochemical cell is shown in Figure S6. The PSD records the voltage output, which corresponds to the curvature changes. LabVIEW software was used to record and calculate the stress associated with curvature using Stoney’s equation. Here, we measure stress-thickness (N m^–1^), which we refer to as stress, using the cantilever bending method through Stoney’s equation
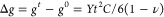
where Δ*g* is the change in surface stress between the front (*g*^t^) and back (*g*^0^) sides of the cantilever, *Y* is Young’s modulus, *t* is the thickness of the cantilever, *v* is the Poisson ratio, and *C* is the curvature of the cantilever.^27,34,36−38,40,65^

For derivative calculations, the surface stress measurements were smoothed using the Savitsky–Golay smoothing function in OriginLab software. The cantilevers were cut to size (5 × 10 mm^2^) using a diamond cutter. The area of the cantilever submerged in solution was approximately 40 mm^2^.

## Supporting Information Available

Additional cyclic voltammograms of Au in 0.1 M KOH in the voltage range of 0.8–1.7 V vs RHE, *pre*-OER region, SFO/Au electrodes with varying high potential from 1.2 to 1.65 V, SrFeO_3−δ_/Au in 0.1 M KOH in the voltage range of 0.8–1.3 V vs RHE, and additional details of electrochemical cell setup for measuring cyclic voltammograms and *in situ* surface stress are included in the SI (PDF)
