# Supplementary material for: Structural Evolution of Ultrathin SrFeO3−δ Films during Oxygen Evolution Reaction Revealed by In Situ Electrochemical Stress Measurements
Source: ACS Appl Energy Mater. 2023 Nov 16;6(23):11882–9. doi: 10.1021/acsaem.3c01805 (PMC10716856; doi:10.1021/acsaem.3c01805)
Supplement: Supplementary file 1 — ae3c01805_si_001.pdf [file ae3c01805_si_001.pdf]

Supporting Information

Structural Evolution of Ultrathin $SrFeO_{3-\delta}$ Films During Oxygen Evolution Reaction Revealed by *In-situ* Electrochemical Stress Measurements

Emily Marquez[1], Kim Hong Keu[1], Andrea Nelson,[2] Benjamin M. Lefler,[3] Steven J. May,[3] and Hadi Tavassol*[1]

[1] Department of Chemistry and Biochemistry, California State University, Long Beach, CA 90840

[2] Department of Physics and Astronomy, California State University, Long Beach, CA 90840

[3] Department of Materials Science and Engineering, Drexel University, PA 19104

Corresponding author's email address: hadi.tavassol@csulb.edu

Keywords: Perovskite oxides, OER, iron oxides, Stress measurements, *in-situ* structural evolutions.

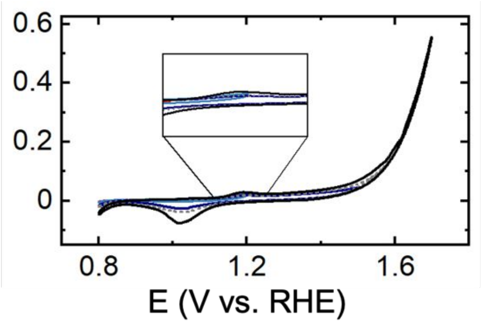

Fig. S1 Cyclic voltammetry of Au in 0.1 M KOH in the voltage range 0.8 V to 1.7 V vs. RHE.

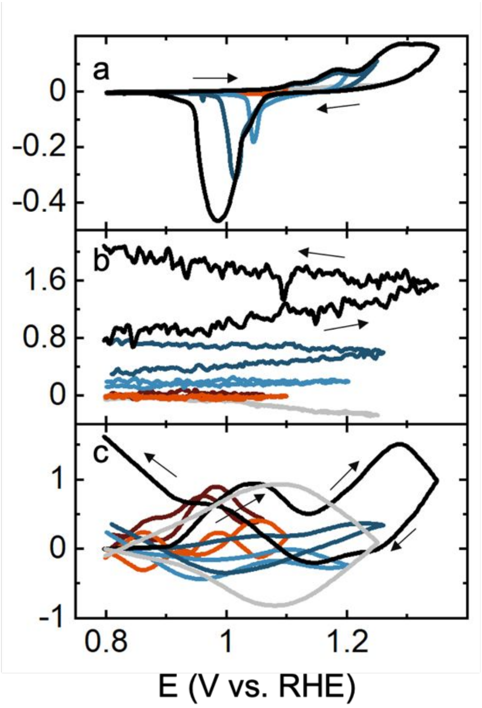

Fig. S2 (a) Cyclic voltammetry of $SrFeO_{3-\delta}$/Au in 0.1 M KOH in the 0.8 V to 1.35 V potential range (*pre*-OER region) at a scan rate of 20 mV s^{-1} (b) corresponding *in situ* stress response, and (c) first derivative of stress with respect to the potential. The data shown in light grey in (b,c) was obtained from Au.

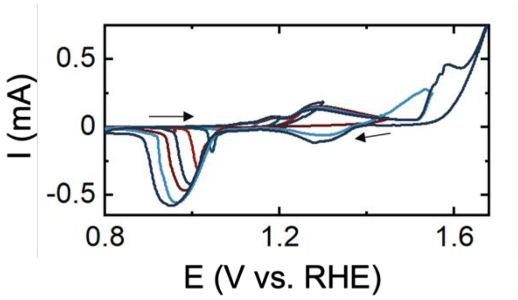

Fig. S3 Cyclic voltammetry analysis of SFO/Au electrodes with varying high potential from 1.2 V to 1.65 V, showing an evolving response with the high potential limit.

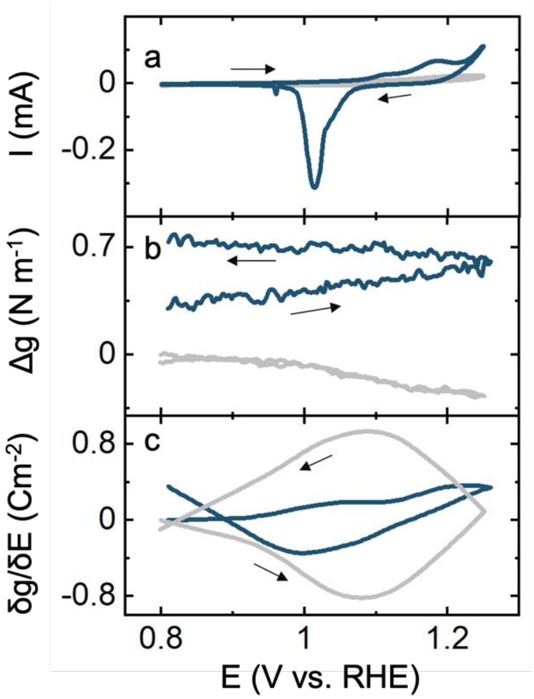

Fig. S4 (a) Cyclic voltammetry of $SrFeO_{3-\delta}$/Au in 0.1 M KOH in the voltage range 0.8 V to 1.3 V vs. RHE. Data corresponding to Au at the high potential limit of 1.25 V is overlayed in light grey. (b) Corresponding *in-situ* stress and (c) first derivative of stress *vs*. potential.

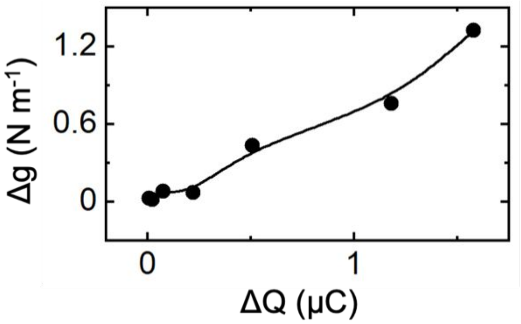

Fig. S5 End-of-cycle residual stress (Δg) as a function of the integrated charge (ΔQ) for $SrFeO_{3-\delta}$/Au in 0.1 M KOH. The charge is calculated by integrating the low potentials from *ca.* 0.95-1.1 V. The integrated range is adjusted as the peak in the CV from Fig. 4 shifts to the left (lower potentials).

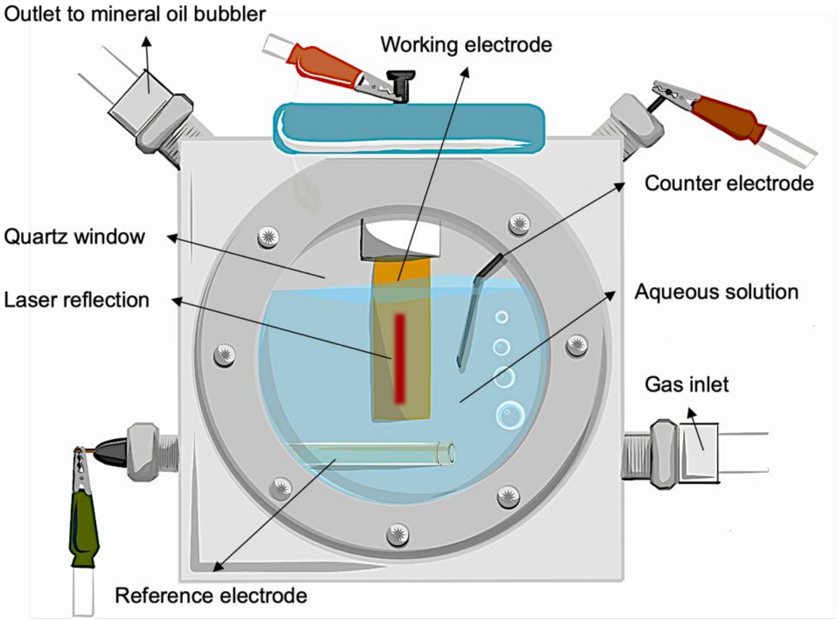

Fig. S6 A schematic of the electrochemical cell set-up for measuring cyclic voltammograms and *in situ* surface stress.
